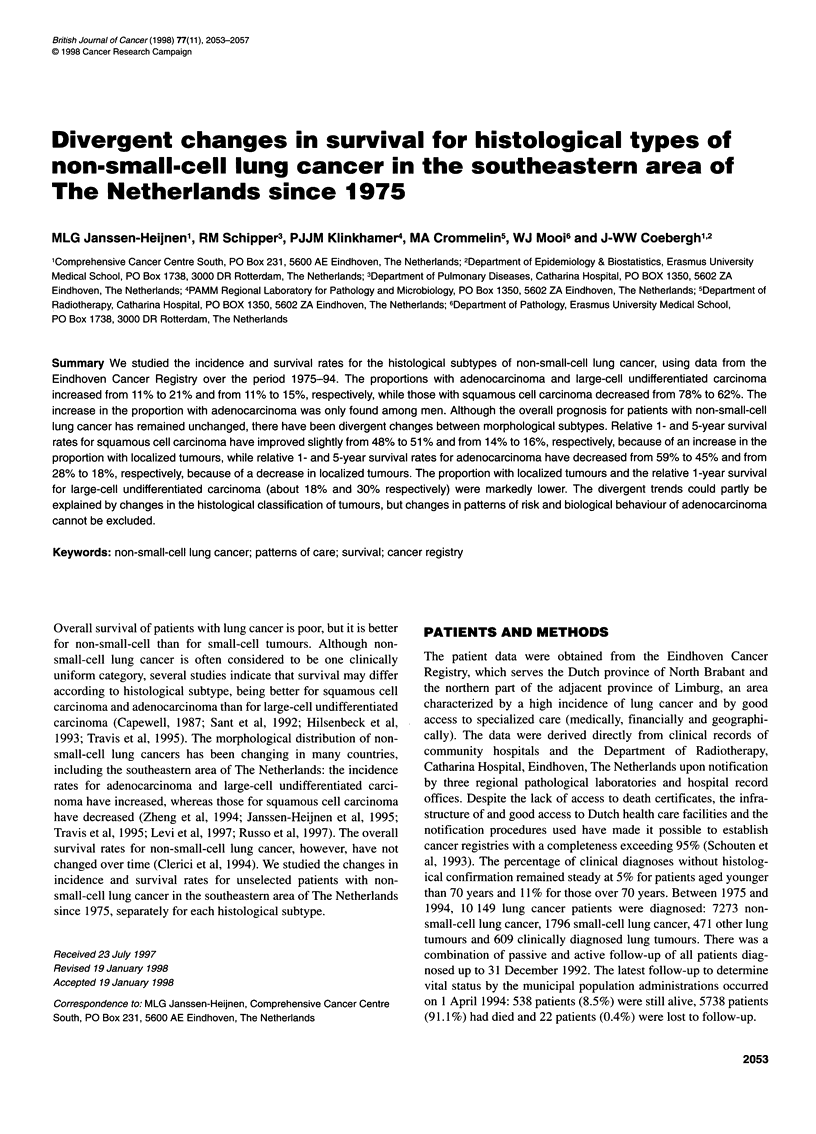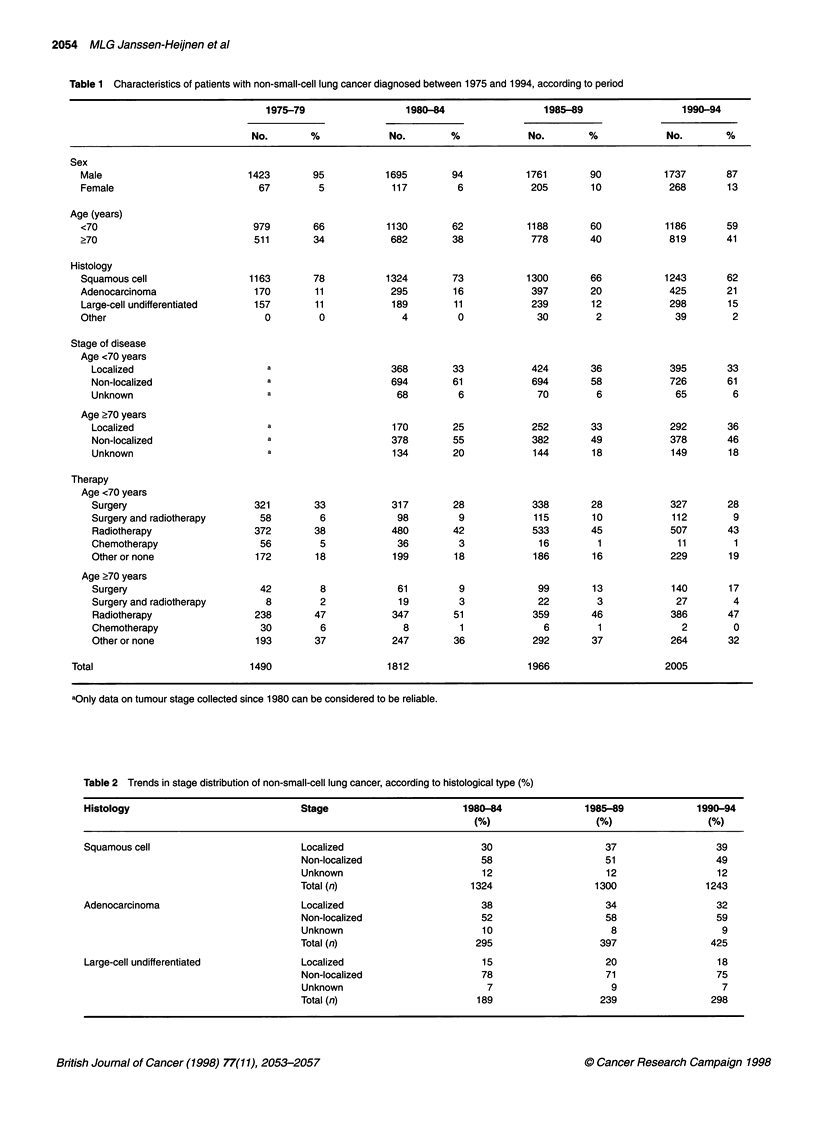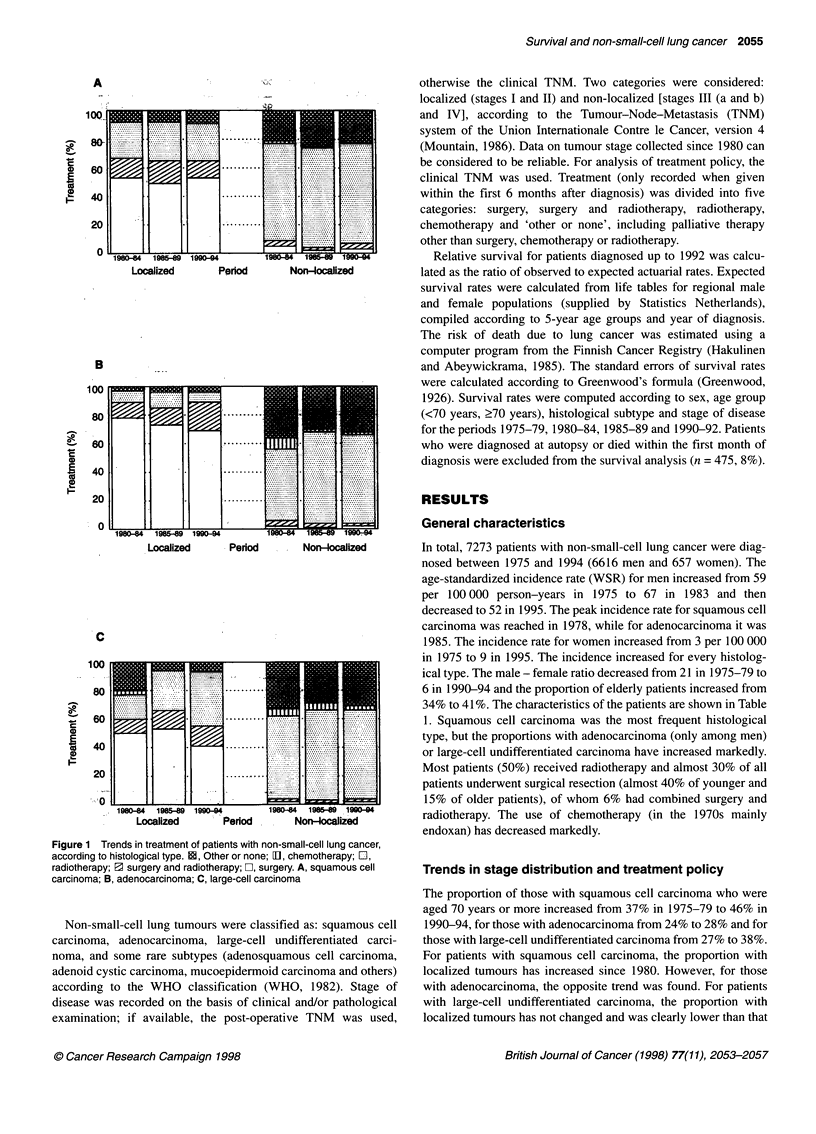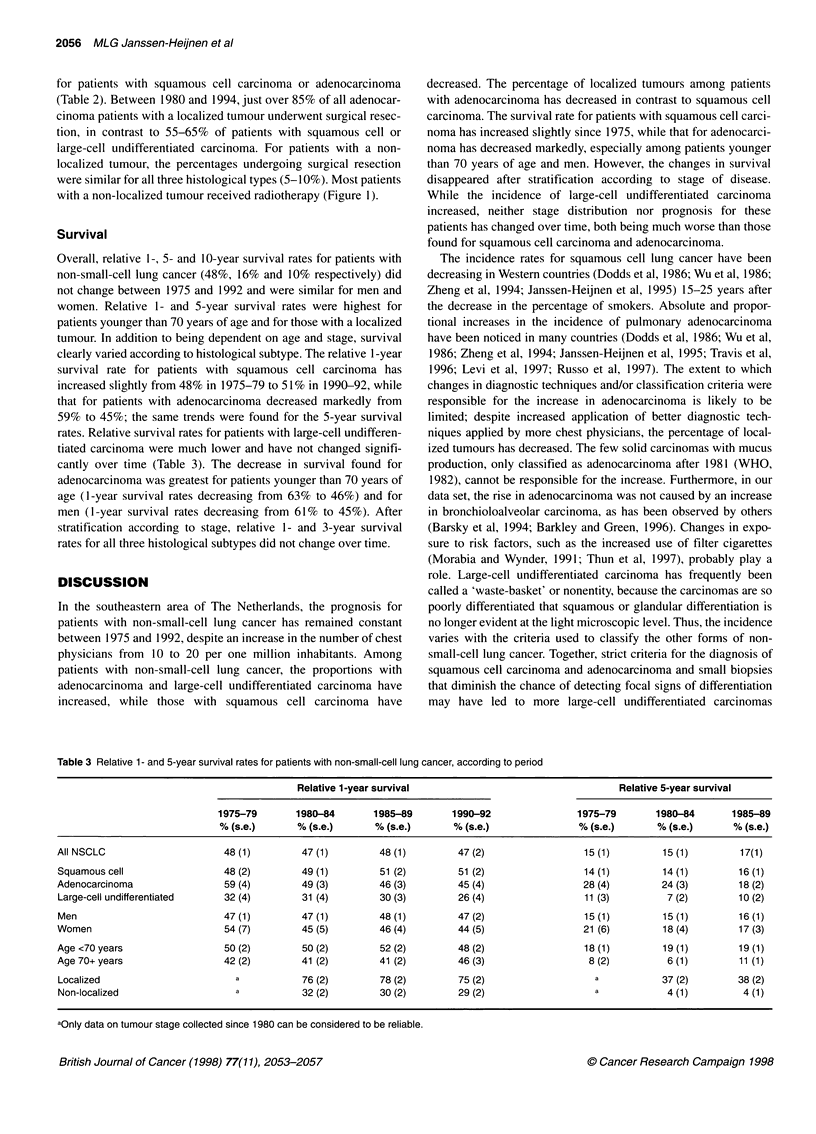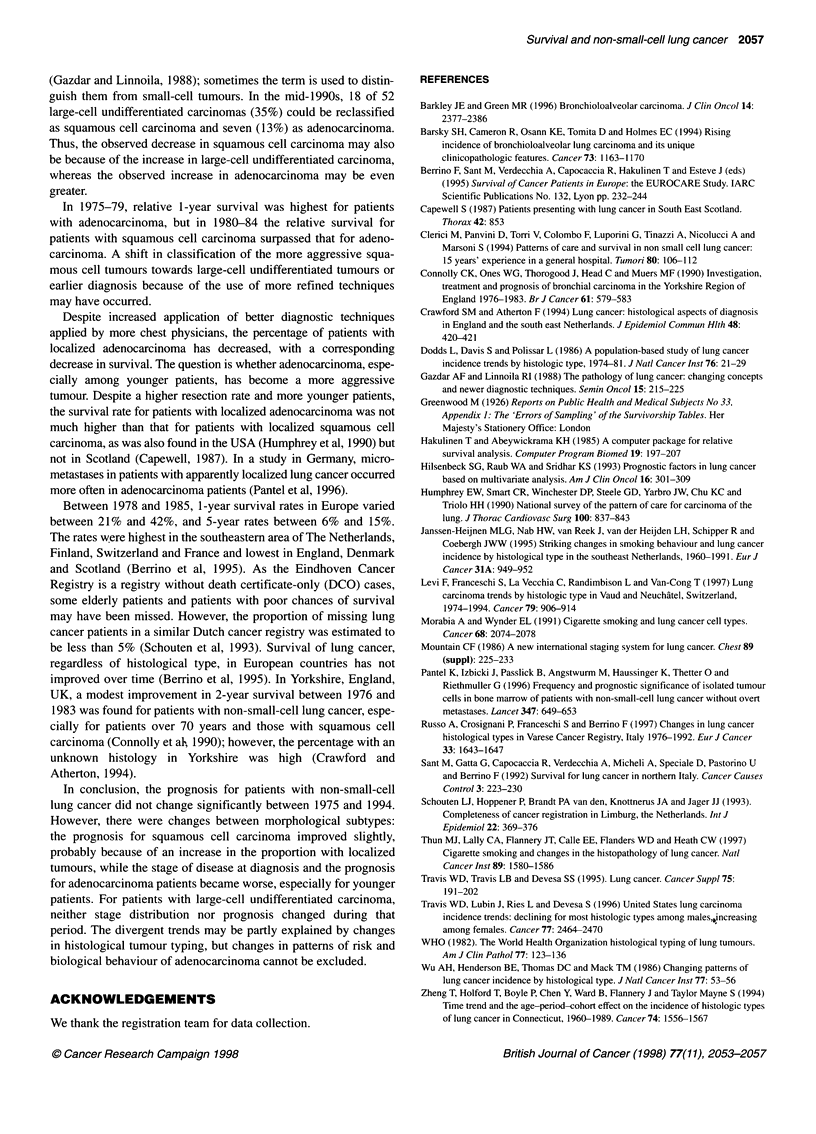# Divergent changes in survival for histological types of non-small-cell lung cancer in the southeastern area of The Netherlands since 1975.

**DOI:** 10.1038/bjc.1998.342

**Published:** 1998-06

**Authors:** M. L. Janssen-Heijnen, R. M. Schipper, P. J. Klinkhamer, M. A. Crommelin, W. J. Mooi, J. W. Coebergh

**Affiliations:** Comprehensive Cancer Centre South, Eindhoven, The Netherlands.

## Abstract

We studied the incidence and survival rates for the histological subtypes of non-small-cell lung cancer, using data from the Eindhoven Cancer Registry over the period 1975-94. The proportions with adenocarcinoma and large-cell undifferentiated carcinoma increased from 11% to 21% and from 11% to 15%, respectively, while those with squamous cell carcinoma decreased from 78% to 62%. The increase in the proportion with adenocarcinoma was only found among men. Although the overall prognosis for patients with non-small-cell lung cancer has remained unchanged, there have been divergent changes between morphological subtypes. Relative 1- and 5-year survival rates for squamous cell carcinoma have improved slightly from 48% to 51% and from 14% to 16%, respectively, because of an increase in the proportion with localized tumours, while relative 1- and 5-year survival rates for adenocarcinoma have decreased from 59% to 45% and from 28% to 18%, respectively, because of a decrease in localized tumours. The proportion with localized tumours and the relative 1-year survival for large-cell undifferentiated carcinoma (about 18% and 30% respectively) were markedly lower. The divergent trends could partly be explained by changes in the histological classification of tumours, but changes in patterns of risk and biological behaviour of adenocarcinoma cannot be excluded.


British Journal of Cancer (1998) 77(11), 2053–2057
© 1998 Cancer Research Campaign

### Divergent changes in survival for histological types of non-small-cell lung cancer in the southeastern area of The Netherlands since 1975

**MLG Janssen-Heijnen[1], RM Schipper[3], PJJM Klinkhamer[4], MA Crommelin[5], WJ Mooi[6] and J-WW Coebergh[1,2]**

[1]Comprehensive Cancer Centre South, PO Box 231, 5600 AE Eindhoven, The Netherlands; [2]Department of Epidemiology & Biostatistics, Erasmus University Medical School, PO Box 1738, 3000 DR Rotterdam, The Netherlands; [3]Department of Pulmonary Diseases, Catharina Hospital, PO BOX 1350, 5602 ZA Eindhoven, The Netherlands; [4]PAMM Regional Laboratory for Pathology and Microbiology, PO Box 1350, 5602 ZA Eindhoven, The Netherlands; [5]Department of Radiotherapy, Catharina Hospital, PO BOX 1350, 5602 ZA Eindhoven, The Netherlands; [6]Department of Pathology, Erasmus University Medical School, PO Box 1738, 3000 DR Rotterdam, The Netherlands

**Summary** We studied the incidence and survival rates for the histological subtypes of non-small-cell lung cancer, using data from the Eindhoven Cancer Registry over the period 1975–94. The proportions with adenocarcinoma and large-cell undifferentiated carcinoma increased from 11% to 21% and from 11% to 15%, respectively, while those with squamous cell carcinoma decreased from 78% to 62%. The increase in the proportion with adenocarcinoma was only found among men. Although the overall prognosis for patients with non-small-cell lung cancer has remained unchanged, there have been divergent changes between morphological subtypes. Relative 1- and 5-year survival rates for squamous cell carcinoma have improved slightly from 48% to 51% and from 14% to 16%, respectively, because of an increase in the proportion with localized tumours, while relative 1- and 5-year survival rates for adenocarcinoma have decreased from 59% to 45% and from 28% to 18%, respectively, because of a decrease in localized tumours. The proportion with localized tumours and the relative 1-year survival for large-cell undifferentiated carcinoma (about 18% and 30% respectively) were markedly lower. The divergent trends could partly be explained by changes in the histological classification of tumours, but changes in patterns of risk and biological behaviour of adenocarcinoma cannot be excluded.

**Keywords:** non-small-cell lung cancer; patterns of care; survival; cancer registry

Overall survival of patients with lung cancer is poor, but it is better for non-small-cell than for small-cell tumours. Although non-small-cell lung cancer is often considered to be one clinically uniform category, several studies indicate that survival may differ according to histological subtype, being better for squamous cell carcinoma and adenocarcinoma than for large-cell undifferentiated carcinoma (Capewell, 1987; Sant et al, 1992; Hilsenbeck et al, 1993; Travis et al, 1995). The morphological distribution of non-small-cell lung cancers has been changing in many countries, including the southeastern area of The Netherlands: the incidence rates for adenocarcinoma and large-cell undifferentiated carcinoma have increased, whereas those for squamous cell carcinoma have decreased (Zheng et al, 1994; Janssen-Heijnen et al, 1995; Travis et al, 1995; Levi et al, 1997; Russo et al, 1997). The overall survival rates for non-small-cell lung cancer, however, have not changed over time (Clerici et al, 1994). We studied the changes in incidence and survival rates for unselected patients with non-small-cell lung cancer in the southeastern area of The Netherlands since 1975, separately for each histological subtype.

*Received 23 July 1997*
*Revised 19 January 1998*
*Accepted 19 January 1998*

*Correspondence to:* MLG Janssen-Heijnen, Comprehensive Cancer Centre South, PO Box 231, 5600 AE Eindhoven, The Netherlands

### PATIENTS AND METHODS

The patient data were obtained from the Eindhoven Cancer Registry, which serves the Dutch province of North Brabant and the northern part of the adjacent province of Limburg, an area characterized by a high incidence of lung cancer and by good access to specialized care (medically, financially and geographically). The data were derived directly from clinical records of community hospitals and the Department of Radiotherapy, Catharina Hospital, Eindhoven, The Netherlands upon notification by three regional pathological laboratories and hospital record offices. Despite the lack of access to death certificates, the infrastructure of and good access to Dutch health care facilities and the notification procedures used have made it possible to establish cancer registries with a completeness exceeding 95% (Schouten et al, 1993). The percentage of clinical diagnoses without histological confirmation remained steady at 5% for patients aged younger than 70 years and 11% for those over 70 years. Between 1975 and 1994, 10 149 lung cancer patients were diagnosed: 7273 non-small-cell lung cancer, 1796 small-cell lung cancer, 471 other lung tumours and 609 clinically diagnosed lung tumours. There was a combination of passive and active follow-up of all patients diagnosed up to 31 December 1992. The latest follow-up to determine vital status by the municipal population administrations occurred on 1 April 1994: 538 patients (8.5%) were still alive, 5738 patients (91.1%) had died and 22 patients (0.4%) were lost to follow-up.

**Table 1** Characteristics of patients with non-small-cell lung cancer diagnosed between 1975 and 1994, according to period

| | 1975–79 | | 1980–84 | | 1985–89 | | 1990–94 | |
|---|---|---|---|---|---|---|---|---|
| | No. | % | No. | % | No. | % | No. | % |
| Sex | | | | | | | | |
| Male | 1423 | 95 | 1695 | 94 | 1761 | 90 | 1737 | 87 |
| Female | 67 | 5 | 117 | 6 | 205 | 10 | 268 | 13 |
| Age (years) | | | | | | | | |
| <70 | 979 | 66 | 1130 | 62 | 1188 | 60 | 1186 | 59 |
| ≥70 | 511 | 34 | 682 | 38 | 778 | 40 | 819 | 41 |
| Histology | | | | | | | | |
| Squamous cell | 1163 | 78 | 1324 | 73 | 1300 | 66 | 1243 | 62 |
| Adenocarcinoma | 170 | 11 | 295 | 16 | 397 | 20 | 425 | 21 |
| Large-cell undifferentiated | 157 | 11 | 189 | 11 | 239 | 12 | 298 | 15 |
| Other | 0 | 0 | 4 | 0 | 30 | 2 | 39 | 2 |
| Stage of disease | | | | | | | | |
| Age <70 years | | | | | | | | |
| Localized | [a] | | 368 | 33 | 424 | 36 | 395 | 33 |
| Non-localized | [a] | | 694 | 61 | 694 | 58 | 726 | 61 |
| Unknown | [a] | | 68 | 6 | 70 | 6 | 65 | 6 |
| Age ≥70 years | | | | | | | | |
| Localized | [a] | | 170 | 25 | 252 | 33 | 292 | 36 |
| Non-localized | [a] | | 378 | 55 | 382 | 49 | 378 | 46 |
| Unknown | [a] | | 134 | 20 | 144 | 18 | 149 | 18 |
| Therapy | | | | | | | | |
| Age <70 years | | | | | | | | |
| Surgery | 321 | 33 | 317 | 28 | 338 | 28 | 327 | 28 |
| Surgery and radiotherapy | 58 | 6 | 98 | 9 | 115 | 10 | 112 | 9 |
| Radiotherapy | 372 | 38 | 480 | 42 | 533 | 45 | 507 | 43 |
| Chemotherapy | 56 | 5 | 36 | 3 | 16 | 1 | 11 | 1 |
| Other or none | 172 | 18 | 199 | 18 | 186 | 16 | 229 | 19 |
| Age ≥70 years | | | | | | | | |
| Surgery | 42 | 8 | 61 | 9 | 99 | 13 | 140 | 17 |
| Surgery and radiotherapy | 8 | 2 | 19 | 3 | 22 | 3 | 27 | 4 |
| Radiotherapy | 238 | 47 | 347 | 51 | 359 | 46 | 386 | 47 |
| Chemotherapy | 30 | 6 | 8 | 1 | 6 | 1 | 2 | 0 |
| Other or none | 193 | 37 | 247 | 36 | 292 | 37 | 264 | 32 |
| Total | 1490 | | 1812 | | 1966 | | 2005 | |

[a]Only data on tumour stage collected since 1980 can be considered to be reliable.

**Table 2** Trends in stage distribution of non-small-cell lung cancer, according to histological type (%)

| Histology | Stage | 1980–84 (%) | 1985–89 (%) | 1990–94 (%) |
|---|---|---|---|---|
| Squamous cell | Localized | 30 | 37 | 39 |
| | Non-localized | 58 | 51 | 49 |
| | Unknown | 12 | 12 | 12 |
| | Total (*n*) | 1324 | 1300 | 1243 |
| Adenocarcinoma | Localized | 38 | 34 | 32 |
| | Non-localized | 52 | 58 | 59 |
| | Unknown | 10 | 8 | 9 |
| | Total (*n*) | 295 | 397 | 425 |
| Large-cell undifferentiated | Localized | 15 | 20 | 18 |
| | Non-localized | 78 | 71 | 75 |
| | Unknown | 7 | 9 | 7 |
| | Total (*n*) | 189 | 239 | 298 |

 © Cancer Research Campaign 1998

**Figure 1** Trends in treatment of patients with non-small-cell lung cancer, according to histological type. ⊠, Other or none; ◫, chemotherapy; ☐, radiotherapy; ▨ surgery and radiotherapy; □, surgery. **A**, squamous cell carcinoma; **B**, adenocarcinoma; **C**, large-cell carcinoma

Non-small-cell lung tumours were classified as: squamous cell carcinoma, adenocarcinoma, large-cell undifferentiated carcinoma, and some rare subtypes (adenosquamous cell carcinoma, adenoid cystic carcinoma, mucoepidermoid carcinoma and others) according to the WHO classification (WHO, 1982). Stage of disease was recorded on the basis of clinical and/or pathological examination; if available, the post-operative TNM was used, otherwise the clinical TNM. Two categories were considered: localized (stages I and II) and non-localized [stages III (a and b) and IV], according to the Tumour–Node–Metastasis (TNM) system of the Union Internationale Contre le Cancer, version 4 (Mountain, 1986). Data on tumour stage collected since 1980 can be considered to be reliable. For analysis of treatment policy, the clinical TNM was used. Treatment (only recorded when given within the first 6 months after diagnosis) was divided into five categories: surgery, surgery and radiotherapy, radiotherapy, chemotherapy and 'other or none', including palliative therapy other than surgery, chemotherapy or radiotherapy.

Relative survival for patients diagnosed up to 1992 was calculated as the ratio of observed to expected actuarial rates. Expected survival rates were calculated from life tables for regional male and female populations (supplied by Statistics Netherlands), compiled according to 5-year age groups and year of diagnosis. The risk of death due to lung cancer was estimated using a computer program from the Finnish Cancer Registry (Hakulinen and Abeywickrama, 1985). The standard errors of survival rates were calculated according to Greenwood's formula (Greenwood, 1926). Survival rates were computed according to sex, age group (<70 years, ≥70 years), histological subtype and stage of disease for the periods 1975–79, 1980–84, 1985–89 and 1990–92. Patients who were diagnosed at autopsy or died within the first month of diagnosis were excluded from the survival analysis ($n$ = 475, 8%).

### RESULTS

### General characteristics

In total, 7273 patients with non-small-cell lung cancer were diagnosed between 1975 and 1994 (6616 men and 657 women). The age-standardized incidence rate (WSR) for men increased from 59 per 100 000 person–years in 1975 to 67 in 1983 and then decreased to 52 in 1995. The peak incidence rate for squamous cell carcinoma was reached in 1978, while for adenocarcinoma it was 1985. The incidence rate for women increased from 3 per 100 000 in 1975 to 9 in 1995. The incidence increased for every histological type. The male – female ratio decreased from 21 in 1975–79 to 6 in 1990–94 and the proportion of elderly patients increased from 34% to 41%. The characteristics of the patients are shown in Table 1. Squamous cell carcinoma was the most frequent histological type, but the proportions with adenocarcinoma (only among men) or large-cell undifferentiated carcinoma have increased markedly. Most patients (50%) received radiotherapy and almost 30% of all patients underwent surgical resection (almost 40% of younger and 15% of older patients), of whom 6% had combined surgery and radiotherapy. The use of chemotherapy (in the 1970s mainly endoxan) has decreased markedly.

### Trends in stage distribution and treatment policy

The proportion of those with squamous cell carcinoma who were aged 70 years or more increased from 37% in 1975–79 to 46% in 1990–94, for those with adenocarcinoma from 24% to 28% and for those with large-cell undifferentiated carcinoma from 27% to 38%. For patients with squamous cell carcinoma, the proportion with localized tumours has increased since 1980. However, for those with adenocarcinoma, the opposite trend was found. For patients with large-cell undifferentiated carcinoma, the proportion with localized tumours has not changed and was clearly lower than that

© Cancer Research Campaign 1998

for patients with squamous cell carcinoma or adenocarcinoma (Table 2). Between 1980 and 1994, just over 85% of all adenocarcinoma patients with a localized tumour underwent surgical resection, in contrast to 55–65% of patients with squamous cell or large-cell undifferentiated carcinoma. For patients with a non-localized tumour, the percentages undergoing surgical resection were similar for all three histological types (5–10%). Most patients with a non-localized tumour received radiotherapy (Figure 1).

### Survival

Overall, relative 1-, 5- and 10-year survival rates for patients with non-small-cell lung cancer (48%, 16% and 10% respectively) did not change between 1975 and 1992 and were similar for men and women. Relative 1- and 5-year survival rates were highest for patients younger than 70 years of age and for those with a localized tumour. In addition to being dependent on age and stage, survival clearly varied according to histological subtype. The relative 1-year survival rate for patients with squamous cell carcinoma has increased slightly from 48% in 1975–79 to 51% in 1990–92, while that for patients with adenocarcinoma decreased markedly from 59% to 45%; the same trends were found for the 5-year survival rates. Relative survival rates for patients with large-cell undifferentiated carcinoma were much lower and have not changed significantly over time (Table 3). The decrease in survival found for adenocarcinoma was greatest for patients younger than 70 years of age (1-year survival rates decreasing from 63% to 46%) and for men (1-year survival rates decreasing from 61% to 45%). After stratification according to stage, relative 1- and 3-year survival rates for all three histological subtypes did not change over time.

### DISCUSSION

In the southeastern area of The Netherlands, the prognosis for patients with non-small-cell lung cancer has remained constant between 1975 and 1992, despite an increase in the number of chest physicians from 10 to 20 per one million inhabitants. Among patients with non-small-cell lung cancer, the proportions with adenocarcinoma and large-cell undifferentiated carcinoma have increased, while those with squamous cell carcinoma have decreased. The percentage of localized tumours among patients with adenocarcinoma has decreased in contrast to squamous cell carcinoma. The survival rate for patients with squamous cell carcinoma has increased slightly since 1975, while that for adenocarcinoma has decreased markedly, especially among patients younger than 70 years of age and men. However, the changes in survival disappeared after stratification according to stage of disease. While the incidence of large-cell undifferentiated carcinoma increased, neither stage distribution nor prognosis for these patients has changed over time, both being much worse than those found for squamous cell carcinoma and adenocarcinoma.

The incidence rates for squamous cell lung cancer have been decreasing in Western countries (Dodds et al, 1986; Wu et al, 1986; Zheng et al, 1994; Janssen-Heijnen et al, 1995) 15–25 years after the decrease in the percentage of smokers. Absolute and proportional increases in the incidence of pulmonary adenocarcinoma have been noticed in many countries (Dodds et al, 1986; Wu et al, 1986; Zheng et al, 1994; Janssen-Heijnen et al, 1995; Travis et al, 1996; Levi et al, 1997; Russo et al, 1997). The extent to which changes in diagnostic techniques and/or classification criteria were responsible for the increase in adenocarcinoma is likely to be limited; despite increased application of better diagnostic techniques applied by more chest physicians, the percentage of localized tumours has decreased. The few solid carcinomas with mucus production, only classified as adenocarcinoma after 1981 (WHO, 1982), cannot be responsible for the increase. Furthermore, in our data set, the rise in adenocarcinoma was not caused by an increase in bronchioloalveolar carcinoma, as has been observed by others (Barsky et al, 1994; Barkley and Green, 1996). Changes in exposure to risk factors, such as the increased use of filter cigarettes (Morabia and Wynder, 1991; Thun et al, 1997), probably play a role. Large-cell undifferentiated carcinoma has frequently been called a 'waste-basket' or nonentity, because the carcinomas are so poorly differentiated that squamous or glandular differentiation is no longer evident at the light microscopic level. Thus, the incidence varies with the criteria used to classify the other forms of non-small-cell lung cancer. Together, strict criteria for the diagnosis of squamous cell carcinoma and adenocarcinoma and small biopsies that diminish the chance of detecting focal signs of differentiation may have led to more large-cell undifferentiated carcinomas

**Table 3** Relative 1- and 5-year survival rates for patients with non-small-cell lung cancer, according to period

| | Relative 1-year survival | | | | Relative 5-year survival | | |
|---|---|---|---|---|---|---|---|
| | 1975–79 % (s.e.) | 1980–84 % (s.e.) | 1985–89 % (s.e.) | 1990–92 % (s.e.) | 1975–79 % (s.e.) | 1980–84 % (s.e.) | 1985–89 % (s.e.) |
| All NSCLC | 48 (1) | 47 (1) | 48 (1) | 47 (2) | 15 (1) | 15 (1) | 17(1) |
| Squamous cell | 48 (2) | 49 (1) | 51 (2) | 51 (2) | 14 (1) | 14 (1) | 16 (1) |
| Adenocarcinoma | 59 (4) | 49 (3) | 46 (3) | 45 (4) | 28 (4) | 24 (3) | 18 (2) |
| Large-cell undifferentiated | 32 (4) | 31 (4) | 30 (3) | 26 (4) | 11 (3) | 7 (2) | 10 (2) |
| Men | 47 (1) | 47 (1) | 48 (1) | 47 (2) | 15 (1) | 15 (1) | 16 (1) |
| Women | 54 (7) | 45 (5) | 46 (4) | 44 (5) | 21 (6) | 18 (4) | 17 (3) |
| Age <70 years | 50 (2) | 50 (2) | 52 (2) | 48 (2) | 18 (1) | 19 (1) | 19 (1) |
| Age 70+ years | 42 (2) | 41 (2) | 41 (2) | 46 (3) | 8 (2) | 6 (1) | 11 (1) |
| Localized | [a] | 76 (2) | 78 (2) | 75 (2) | [a] | 37 (2) | 38 (2) |
| Non-localized | [a] | 32 (2) | 30 (2) | 29 (2) | [a] | 4 (1) | 4 (1) |

[a]Only data on tumour stage collected since 1980 can be considered to be reliable.

*© Cancer Research Campaign 1998*

(Gazdar and Linnoila, 1988); sometimes the term is used to distinguish them from small-cell tumours. In the mid-1990s, 18 of 52 large-cell undifferentiated carcinomas (35%) could be reclassified as squamous cell carcinoma and seven (13%) as adenocarcinoma. Thus, the observed decrease in squamous cell carcinoma may also be because of the increase in large-cell undifferentiated carcinoma, whereas the observed increase in adenocarcinoma may be even greater.

In 1975–79, relative 1-year survival was highest for patients with adenocarcinoma, but in 1980–84 the relative survival for patients with squamous cell carcinoma surpassed that for adenocarcinoma. A shift in classification of the more aggressive squamous cell tumours towards large-cell undifferentiated tumours or earlier diagnosis because of the use of more refined techniques may have occurred.

Despite increased application of better diagnostic techniques applied by more chest physicians, the percentage of patients with localized adenocarcinoma has decreased, with a corresponding decrease in survival. The question is whether adenocarcinoma, especially among younger patients, has become a more aggressive tumour. Despite a higher resection rate and more younger patients, the survival rate for patients with localized adenocarcinoma was not much higher than that for patients with localized squamous cell carcinoma, as was also found in the USA (Humphrey et al, 1990) but not in Scotland (Capewell, 1987). In a study in Germany, micrometastases in patients with apparently localized lung cancer occurred more often in adenocarcinoma patients (Pantel et al, 1996).

Between 1978 and 1985, 1-year survival rates in Europe varied between 21% and 42%, and 5-year rates between 6% and 15%. The rates were highest in the southeastern area of The Netherlands, Finland, Switzerland and France and lowest in England, Denmark and Scotland (Berrino et al, 1995). As the Eindhoven Cancer Registry is a registry without death certificate-only (DCO) cases, some elderly patients and patients with poor chances of survival may have been missed. However, the proportion of missing lung cancer patients in a similar Dutch cancer registry was estimated to be less than 5% (Schouten et al, 1993). Survival of lung cancer, regardless of histological type, in European countries has not improved over time (Berrino et al, 1995). In Yorkshire, England, UK, a modest improvement in 2-year survival between 1976 and 1983 was found for patients with non-small-cell lung cancer, especially for patients over 70 years and those with squamous cell carcinoma (Connolly et al, 1990); however, the percentage with an unknown histology in Yorkshire was high (Crawford and Atherton, 1994).

In conclusion, the prognosis for patients with non-small-cell lung cancer did not change significantly between 1975 and 1994. However, there were changes between morphological subtypes: the prognosis for squamous cell carcinoma improved slightly, probably because of an increase in the proportion with localized tumours, while the stage of disease at diagnosis and the prognosis for adenocarcinoma patients became worse, especially for younger patients. For patients with large-cell undifferentiated carcinoma, neither stage distribution nor prognosis changed during that period. The divergent trends may be partly explained by changes in histological tumour typing, but changes in patterns of risk and biological behaviour of adenocarcinoma cannot be excluded.

### ACKNOWLEDGEMENTS

We thank the registration team for data collection.

### REFERENCES

Barkley JE and Green MR (1996) Bronchioloalveolar carcinoma. *J Clin Oncol* **14**: 2377–2386

Barsky SH, Cameron R, Osann KE, Tomita D and Holmes EC (1994) Rising incidence of bronchioloalveolar lung carcinoma and its unique clinicopathologic features. *Cancer* **73**: 1163–1170

Berrino F, Sant M, Verdecchia A, Capocaccia R, Hakulinen T and Esteve J (eds) (1995) *Survival of Cancer Patients in Europe*: the EUROCARE Study. IARC Scientific Publications No. 132, Lyon pp. 232–244

Capewell S (1987) Patients presenting with lung cancer in South East Scotland. *Thorax* **42**: 853

Clerici M, Panvini D, Torri V, Colombo F, Luporini G, Tinazzi A, Nicolucci A and Marsoni S (1994) Patterns of care and survival in non small cell lung cancer: 15 years' experience in a general hospital. *Tumori* **80**: 106–112

Connolly CK, Ones WG, Thorogood J, Head C and Muers MF (1990) Investigation, treatment and prognosis of bronchial carcinoma in the Yorkshire Region of England 1976–1983. *Br J Cancer* **61**: 579–583

Crawford SM and Atherton F (1994) Lung cancer: histological aspects of diagnosis in England and the south east Netherlands. *J Epidemiol Commun Hlth* **48**: 420–421

Dodds L, Davis S and Polissar L (1986) A population-based study of lung cancer incidence trends by histologic type, 1974–81. *J Natl Cancer Inst* **76**: 21–29

Gazdar AF and Linnoila RI (1988) The pathology of lung cancer: changing concepts and newer diagnostic techniques. *Semin Oncol* **15**: 215–225

Greenwood M (1926) *Reports on Public Health and Medical Subjects No 33, Appendix 1: The 'Errors of Sampling' of the Survivorship Tables*. Her Majesty's Stationery Office: London

Hakulinen T and Abeywickrama KH (1985) A computer package for relative survival analysis. *Computer Program Biomed* **19**: 197–207

Hilsenbeck SG, Raub WA and Sridhar KS (1993) Prognostic factors in lung cancer based on multivariate analysis. *Am J Clin Oncol* **16**: 301–309

Humphrey EW, Smart CR, Winchester DP, Steele GD, Yarbro JW, Chu KC and Triolo HH (1990) National survey of the pattern of care for carcinoma of the lung. *J Thorac Cardiovasc Surg* **100**: 837–843

Janssen-Heijnen MLG, Nab HW, van Reek J, van der Heijden LH, Schipper R and Coebergh JWW (1995) Striking changes in smoking behaviour and lung cancer incidence by histological type in the southeast Netherlands, 1960–1991. *Eur J Cancer* **31A**: 949–952

Levi F, Franceschi S, La Vecchia C, Randimbison L and Van-Cong T (1997) Lung carcinoma trends by histologic type in Vaud and Neuchâtel, Switzerland, 1974–1994. *Cancer* **79**: 906–914

Morabia A and Wynder EL (1991) Cigarette smoking and lung cancer cell types. *Cancer* **68**: 2074–2078

Mountain CF (1986) A new international staging system for lung cancer. *Chest* **89 (suppl)**: 225–233

Pantel K, Izbicki J, Passlick B, Angstwurm M, Haussinger K, Thetter O and Riethmuller G (1996) Frequency and prognostic significance of isolated tumour cells in bone marrow of patients with non-small-cell lung cancer without overt metastases. *Lancet* **347**: 649–653

Russo A, Crosignani P, Franceschi S and Berrino F (1997) Changes in lung cancer histological types in Varese Cancer Registry, Italy 1976–1992. *Eur J Cancer* **33**: 1643–1647

Sant M, Gatta G, Capocaccia R, Verdecchia A, Micheli A, Speciale D, Pastorino U and Berrino F (1992) Survival for lung cancer in northern Italy. *Cancer Causes Control* **3**: 223–230

Schouten LJ, Hoppener P, Brandt PA van den, Knottnerus JA and Jager JJ (1993). Completeness of cancer registration in Limburg, the Netherlands. *Int J Epidemiol* **22**: 369–376

Thun MJ, Lally CA, Flannery JT, Calle EE, Flanders WD and Heath CW (1997) Cigarette smoking and changes in the histopathology of lung cancer. *Natl Cancer Inst* **89**: 1580–1586

Travis WD, Travis LB and Devesa SS (1995). Lung cancer. *Cancer Suppl* **75**: 191–202

Travis WD, Lubin J, Ries L and Devesa S (1996) United States lung carcinoma incidence trends: declining for most histologic types among males, increasing among females. *Cancer* **77**: 2464–2470

WHO (1982). The World Health Organization histological typing of lung tumours. *Am J Clin Pathol* **77**: 123–136

Wu AH, Henderson BE, Thomas DC and Mack TM (1986) Changing patterns of lung cancer incidence by histological type. *J Natl Cancer Inst* **77**: 53–56

Zheng T, Holford T, Boyle P, Chen Y, Ward B, Flannery J and Taylor Mayne S (1994) Time trend and the age–period–cohort effect on the incidence of histologic types of lung cancer in Connecticut, 1960–1989. *Cancer* **74**: 1556–1567

© Cancer Research Campaign 1998